# Manipulation of microvillar proteins during *Salmonella enterica* invasion results in brush border effacement and actin remodeling

**DOI:** 10.3389/fcimb.2023.1137062

**Published:** 2023-03-02

**Authors:** Alfonso Felipe-López, Nicole Hansmeier, Claudia Danzer, Michael Hensel

**Affiliations:** ^1^ Abt. Mikrobiologie, Universität Osnabrück, Osnabrück, Germany; ^2^ Mikrobiologisches Institut, Universitätsklinikum Erlangen, Erlangen, Germany

**Keywords:** type III secretion system, brush border, F-actin dynamics, cell invasion, polarized epithelial cell

## Abstract

Enterocyte invasion by the gastrointestinal pathogen *Salmonella enterica* is accompanied by loss of brush border and massive remodeling of the actin cytoskeleton, leading to microvilli effacement and formation of membrane ruffles. These manipulations are mediated by effector proteins translocated by the *Salmonella* Pathogenicity Island 1-encoded type III secretion system (SPI1-T3SS). To unravel the mechanisms of microvilli effacement and contribution of SPI1-T3SS effector proteins, the dynamics of host-pathogen interactions was analyzed using live cell imaging (LCI) of polarized epithelial cells (PEC) expressing LifeAct-GFP. PEC were infected with *S. enterica* wild-type and mutant strains with defined defects in SPI1-T3SS effector proteins, and pharmacological inhibition of actin assembly were applied. We identified that microvilli effacement involves two distinct mechanisms: i) F-actin depolymerization mediated by villin and ii), the consumption of cytoplasmic G-actin by formation of membrane ruffles. By analyzing the contribution of individual SPI1-T3SS effector proteins, we demonstrate that SopE dominantly triggers microvilli effacement and formation of membrane ruffles. Furthermore, SopE *via* Rac1 indirectly manipulates villin, which culminates in F-actin depolymerization. Collectively, these results indicate that SopE has dual functions during F-actin remodeling in PEC. While SopE-Rac1 triggers F-actin polymerization and ruffle formation, activation of PLCγ and villin by SopE depolymerizes F-actin in PEC. These results demonstrate the key role of SopE in destruction of the intestinal barrier during intestinal infection by *Salmonella*.

## Introduction

The intestinal epithelium provides an efficient barrier between the sterile tissue of mammalian organisms and the intestinal microbiota, as well as exogenous pathogens (Rogers et al., 2022). Despite the large surface area of the intestinal epithelium, a single cell layer of enterocytes is sufficient to fulfil the uptake of nutrients, secretion of material into the lumen, and protect the organism from intestinal pathogens. This layer is also part of the mucosal immune system.

Enterocytes are polarized epithelial cells (PEC) with unique cellular architecture, i.e. a dense array of microvilli (MV) on the apical side, and presence of cell contacts maintained by tight junctions between cells in the mucosa (Crawley et al., 2014). The brush border generates a massive extension of the intestinal surface important for nutrient uptake, but also acts as an important barrier for protection of underlying sterile tissue against infections.

Various bacterial pathogens have evolved strategies to interfere with the barrier function of the brush border, including the production of toxins, localized colonization and penetration of the epithelial layer by invasion (Sansonetti, 2004). *Salmonella enterica* is a gastrointestinal pathogen with the ability to invade enterocytes, and effacement of the brush border during *Salmonella* invasion was reported (Takeuchi, 1967; Finlay et al., 1988; Gerlach et al., 2008). During analyses of PEC invasion by *Salmonella*, we observed a number of remarkable alterations to infected host cells: i) invasion results in the complete loss of MV (Gerlach et al., 2008) and ii), invasion is more efficient in polarized cells compared to non-polarized host cells (Hölzer and Hensel, 2012). However, the molecular mechanisms deployed by *Salmonella* to destroy brush border integrity have not been investigated in detail.

MV consists of F-actin bundled by villin, EPS8 L3 and fimbrin (plastin I), and anchored to the membrane by myosin 1a, Ezrin, and EBP50 (Brown and McKnight, 2010). The microvillar structure is mainly regulated by the intracellular Ca^2+^ concentration and calmodulin (Bretscher and Weber, 1980; Wolenski et al., 1993; Lin et al., 1994; Brown and McKnight, 2010; Lange, 2011). MV are dynamic structures with a half-life of 10 min. with continuous retraction and rebuilding (Gorelik et al., 2003). This turnover is governed by polymerization of F-actin at the tip of MV and depolymerization at the terminal web (Pollard and Mooseker, 1981; Rzadzinska et al., 2004). The unique arrangement of microvillar proteins and the F-actin bundles results in a crystal-like structure in MV that isolates molecules at the tips of MV from the cell body (Brown and McKnight, 2010; Lange, 2011). Although the microvillar proteins would impede the free transport of actin monomers to the MV tips, myosins can work as shuttle proteins in a manner similar to the transport observed for myosin XVb in stereocilia (Rzadzinska et al., 2004). Further evidence supporting the role of myosins as shuttle proteins was observed in lamellipodia of endothelial cells, where myosin 1c transports G-actin to the ridge of the lamellipodia (Rzadzinska et al., 2004; Mcconnell et al., 2009; Fan et al., 2012).

*Salmonella* invasion is mediated by action of various effector proteins translocated into the host cell by a type III secretion system (T3SS) encoded by genes within *Salmonella* Pathogenicity Island 1 (SPI1). The translocation of SipA, SipC, SopB, SopE, and SopE2 results in remodeling of the F-actin cytoskeleton that concludes in ruffle formation and *Salmonella* engulfment (reviewed in Hume et al., 2017; Fattinger et al., 2021). The *Salmonella* guanine nucleotide exchange factor (GEF) SopE and its homologue SopE2 (Hardt et al., 1998b; Stender et al., 2000) target Rac1, thus triggering F-actin polymerization, membrane ruffling and macropinocytosis by recruitment of WASP and Arp2/3 and other nucleator proteins (Hanisch et al., 2010; Hanisch et al., 2011). Cdc42, another Rho GTPase able to induce F-actin polymerization is also activated by SopE2 and, in lesser extent, SopE (Stender et al., 2000; Friebel et al., 2001). SipA together with SipC nucleates F-actin polymerization (Hayward and Koronakis, 1999; Zhou et al., 1999) and restrict the formation of actin tails during ruffle formation (Perrett and Jepson, 2009). SopB increases the level of inositol 1,3,5 tri-phosphate (Norris et al., 1998), activates Akt (Steele-Mortimer et al., 2000), and plays a role in the biogenesis of the *Salmonella*-containing vacuole during intracellular life of *Salmonella* (Hernandez et al., 2004).

While the functions of these various effector proteins in manipulation of the actin cytoskeleton during *Salmonella* invasion were studied in detail, their roles in interfering with the brush border of PEC are less well understood. In this study, we set out to identify the factors required for interfering with the barrier function of epithelial cells and brush border organization. Here we demonstrate that SopE is sufficient to induce brush border effacement by disruption of F-actin in MV, resulting in an increased G-actin pool that supplies F-actin formation in membrane ruffles. We propose an amplification loop for remodeling of the actin cytoskeleton triggered by *Salmonella.*

## Results

### Invasion of polarized epithelial cells by STM induces brush border effacement and reticular F-actin formation

**Figure 1:**
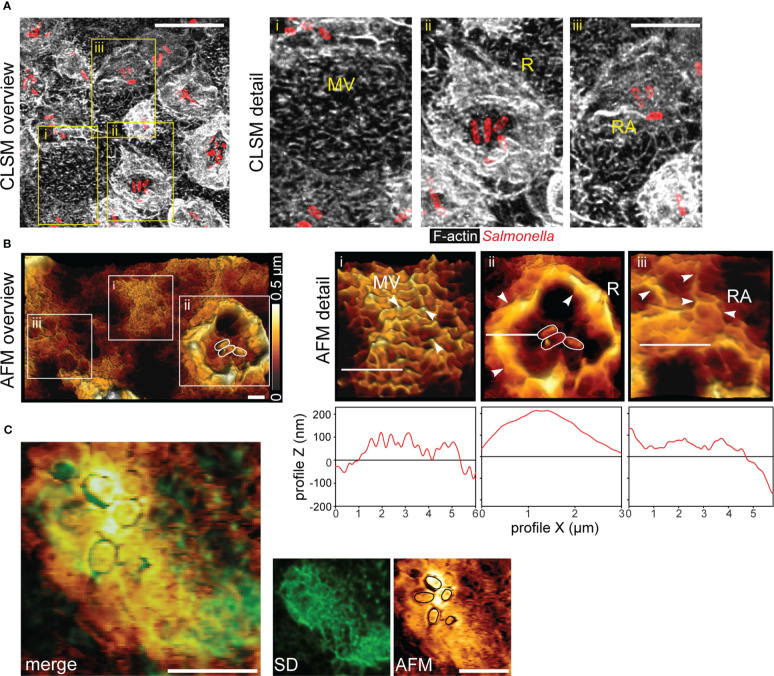
*Salmonella* invasion causes loss of the brush border architecture in polarized epithelial cells by reorganization of F-actin. **(A)** STM disrupts the brush border of MDCK cells. MDCK cells were infected with STM wild type (WT) (red) and fixed 25 min post infection (p.i.), labeled with Phalloidin-Alexa488 (white) and analyzed by confocal laser scanning microscopy (CLSM). Micrographs show an overview of the infected monolayer and boxes indicate (i) cells retaining normal architecture of the brush border microvilli (MV), (ii) membrane ruffle (R) morphology and disruption of MV, and (iii) reticular F-actin (RA) appearing after bacterial internalization. See also **Figure S1** for micrographs of single Z-planes. **(B)** Atomic force microscopy (AFM) analyses of 3D topography. An overview of apical topography of a monolayer of MDCK cells after STM infection is shown and sample height is indicated by a heat map. White boxes indicate positions of detail micrographs of distinct MV (i), R (ii) and RA (iii) phenotypes (highlighted by arrowheads). White bars indicate the positions of height profile analyses plotted below detail micrographs. **(C)** Reticular F-actin formation leads to remodeling of the apical membrane of PEC. MDCK Lifeact-eGFP cells were infected with STM, fixed 1 h p.i. The same area was imaged by spinning disc microscopy (SDM) and AFM. Positions of adherent STM cells are indicated (black lines). Merge of both images indicates that F-actin (green) is fully underlying the reticular surface structures of the apical membrane. Scale bars: 10 µm **(A)**, 2 µm **(B)**, and 5 µm **(C)**.

Previous observation showed that *S. enterica* serovar Typhimurium (STM) is able to invade PEC from the apical side. Apical invasion is accompanied by massive remodeling of the actin cytoskeleton and formation of membrane ruffles. Here we investigated the fate of the host cell brush border during STM invasion. We infected PEC line MDCK with STM wild-type (WT) bacteria. Cells not associated with STM maintained the typical finger-like appearance of the brush border MV (**Figures 1A, B**, detail i). For infected cells we observed following modification of the apical side. a) Cells in contact with STM with formation of massive membrane ruffles and loss of MV (**Figures 1A, B**, detail ii). b) Cells with intracellular STM had neither ruffles nor brush border, but showed prominent reticular structures extending over the entire apical cell side (**Figures 1A, B**, detail iii). Fluorescence microscopy (FM) of Lifeact-eGFP transfected MDCK cells was performed to visualize F-actin and atomic force microscopy (AFM) to profile surface topologies (**Figure 1C**). Correlation of FM and AFM modalities indicated that elevated reticular structures fully co-localized with F-actin. Thus, we termed this morphotype ‘reticular actin’. These observations define STM as a pathogen inducing MV effacement.

### Effector protein SopE is sufficient for polarized cell invasion, microvilli effacement, and reticular F-actin formation

**Figure 2:**
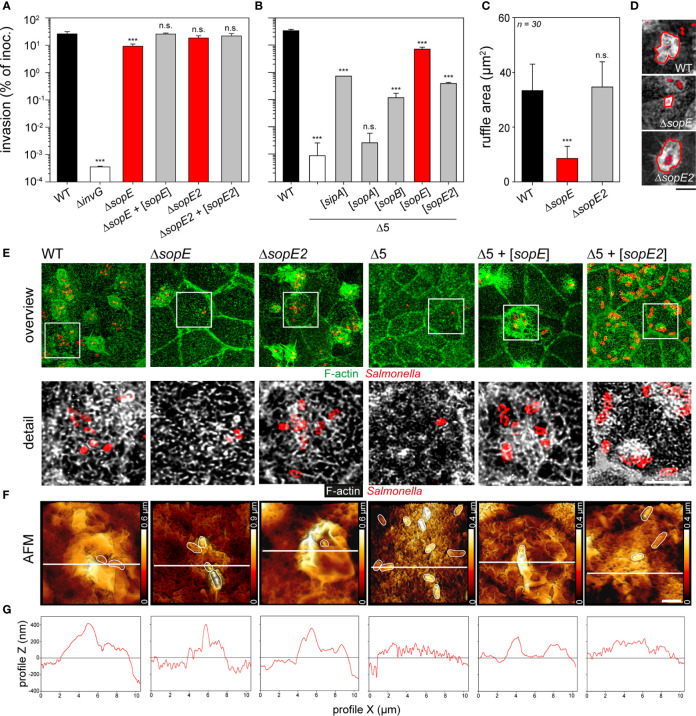
Microvilli effacement and reticular F-actin formation are caused by the translocation of SopE. **(A, B)** Polarized MDCK monolayers were infected with STM WT and various mutant strains for 25 min. After washing, non-internalized bacteria were killed by incubation with medium containing 100 µg x ml^-1^ gentamicin for 1 h. Cells were lysed and internalized bacteria were quantified by plating onto agar plates. Invasion rates are expressed as percentage of the inoculum applied. **(A)** Invasion of MDCK cells by WT, mutant strains deficient in single effector genes, and plasmid-complemented mutant strains indicated by [*sopE*] and [*sopE2*]. **(B)** Invasion of MDCK cells by mutant strain Δ5 lacking *sipA sopA sopB sopE sopE2*, and Δ5 complemented with plasmids expressing single effector genes. For tests of further effectors, and invasion of human C2BBe1 and non-polarized HeLa cells, see **Figure S2**. For microscopy, MDCK cells were infected for 15 min **(C, D)** or 25 min **(E, F)** with the indicated strains expressing mTagRFP (red). Subsequently, cells were washed, fixed with PFA, permeabilized with 0.5% Triton X-100 and stained with Phalloidin-Alexa488 (green or white). Micrographs of infected C2BBe1 and HeLa cells are presented in **Figure S2C**) Deletion of *sopE* results in decreased ruffle areas. Ruffle areas were determined using maximal intensity projection (MIP) from CLSM micrographs to obtain the maximal area of each element. Areas of at least 30 ruffles per strain were quantified after manually outlining the ruffle front. **(D)** Representative images used to quantify ruffle areas. Scale bar, 5 µm. **(E)** Translocation of SopE results in MV effacement and formation of reticular F-actin. MIP images of representative cells after infection with various strains are shown and white boxes indicate the detailed positions of detail micrographs shown below (F-actin, green or white, *Salmonella*, red). Scale bars, 15 and 5 µm for overview and details, respectively. **(F)** 3D AFM topographies of apical surfaces of MDCK cells infected with various strains were generated as in **Figure 1**. White lines indicate positions of height scans shown in **(G)**. Scale bars, 2 µm. One-way ANOVA was applied for statistical analysis and results are indicated as n.s., not significant; ***P < 0.001.

We set out to identify the effector protein(s) responsible for the remodeling of the apical F-actin cytoskeleton of PEC. Invasion of MDCK or C2BBe1 cells by STM WT or mutant strains lacking SPI1-T3SS effector proteins SopE, SopE2, SopA, SopB or SipA was quantified (**Figures 2A**, **S2A, C**). Lack of SopE reduced invasiveness about 3-fold (26.0 ± 5.3% *vs* 9.2 ± 2.1% for WT and Δ*sopE*, respectively) and reduced areas of membrane ruffles were determined (33.4 ± 9.7 µm^2^ *vs.* 8.5 ± 4.4 µm^2^ for WT and Δ*sopE*, respectively) (**Figures 2A, C** **)**. Lack of SopE2, SipA, SopA, or SopB had similar small, or no effects on membrane ruffling and invasion of MDCK and C2BBe1 cells (**Figures 2A, D, E** **;** **S2A, B, C, D, E**). Since previous molecular analyses of SPI1-T3SS effector proteins revealed partially redundant functions in host cell F-actin manipulation (Hardt et al., 1998b; Zhou et al., 1999; Stender et al., 2000; Zhou et al., 2001; Hernandez et al., 2004; Boyle et al., 2006; Patel and Galan, 2006), we deployed a reductionist approach to monitor the contribution of each effector protein to the MV effacement. A mutant strain termed STM Δ5 lacking *sipA, sopA, sopB, sopE*, and *sopE2* was still able to adhere to the apical side of host cells, but neither induced membrane ruffling, nor MV effacement (**Figures 2E, F** **)**. STM Δ5 was more than 10,000-fold reduced in invasion of PEC (**Figures 2B, E, F** **)**, comparable to STM Δ*invG* lacking a functional SPI1-T3SS. We next complemented STM Δ5 with low-copy number plasmids for expression of single effectors, and determined invasion (**Figure 2B**). Complementation with *sipA*, *sopB*, *sopE*, or *sopE2*, but not *sopA*, each resulted in polarized cell invasion significantly higher than that of Δ5. Complementation by *sopE* was sufficient to restore invasion of MDCK cells to 21.0% of WT invasion, as well as membrane ruffling **(** **Figures 2E, F** **)**. Complementation with *sopE2* resulted in only 1.2% of WT invasion (**(** **Figure 2B**), and limited F-actin accumulations were associated with bacteria **(** **Figures 2E, F** **)**. Cells infected with WT, Δ*sopE2*, or Δ5 + [*sopE*] strains showed loss of MV and the presence of reticular F-actin. Invasion phenotypes were similar in the human cell line C2BBe1 (**Figures S2C, D, E**), but far less pronounced in the non-polarized cell line HeLa (**Figures 2C, D, F, G** **)**. Compared to polarized cells, the invasion of non-polarized cells by STM was much lower **(** **Figures 2A, B** vs. **Figures S2D, G**).

Altogether, these results demonstrate that SopE as effector protein with GEF function is sufficient to trigger invasion of STM, to induce MV effacement, and formation of reticular F-actin networks. Although the contribution to invasion is similar in polarized and non-polarized epithelial cell lines, the effects of SopE on apical surface morphology are unique for polarized epithelial cells.

### SopE is sufficient for destruction of barrier functions of polarized epithelial monolayers

**Figure 3:**
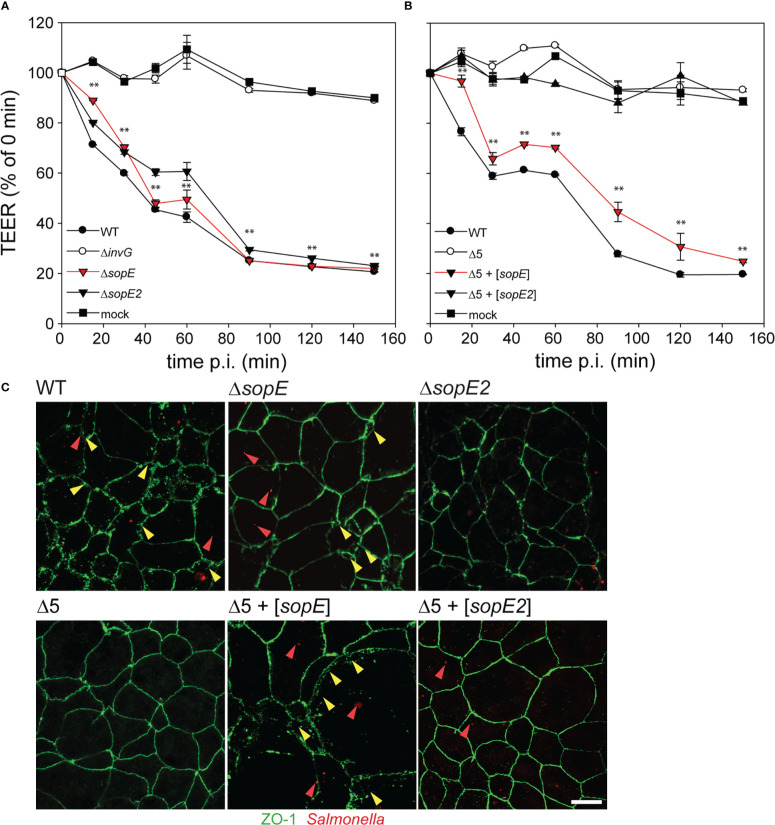
Translocation of SopE is sufficient to breach epithelial barriers. C2BBe1 cells were seeded on polycarbonate filters with a pore size of 0.4 µm in transwell inserts, and incubated for 10-15 d in order to allow the formation of polarized monolayers, as indicated by increase of transepithelial electrical resistance (TEER) to 500-700 Ω per well. **(A)** Cells were infected with STM WT and various mutant strains lacking single effector genes *sopE* or *sopE2*, or defective in SPI1-T3SS (Δ*invG*) at MOI 50. **(B)** Cells were infected with the Δ5 strain without or with plasmids for expression of *sopE* or *sopE2*. The TEER was determined over 2.5 h. The effect on epithelial barrier function is expressed as percentage of TEER immediately prior infection. Means and standard errors are shown (n = 2). One-way ANOVA was applied for statistical analysis and is indicated as for : **P < 0.01. **(C)** After infection with various strains expressing mCherry (red), cells were fixed and tight junctions (TJ) were immuno-stained for ZO-1 (green). Red and yellow arrowheads indicate bacteria and regions of destruction of TJ, respectively. Scale bar, 10 µm.

F-actin is also involved in maintaining the tight junction organization of cells in epithelial tissues (Eaton et al., 1995; Gopalakrishnan et al., 2002). We hypothesized that the effect of SopE on the actin cytoskeleton might affect the barrier function of polarized epithelia. As indicators of epithelial barrier integrity, the transepithelial electrical resistance (TEER) and distribution of tight-junctions (TJ) protein ZO-1 were analyzed in response to STM infection of C2BBe1 cells (**Figure 3**). Infection with STM WT resulted in loss of epithelial barrier function as indicated by loss of TEER, while STM Δ*invG* had no effect on barrier function (**Figure 3A**). Mutant strains only lacking single effectors Δ*sopE* or Δ*sopE2* showed similar reduction in TEER as WT infection. Infection with STM Δ5 + [*sopE*] resulted in TEER decrease similar to the WT strain, whereas infection by STM Δ5 + [*sopE2*] (**Figure 3B**) or other effectors (data not shown) did not result in epithelial barrier damage. The effects of various strains on TJ integrity as determined by continuity of ZO-1 distribution were in line with TEER reduction by various mutant strains (**Figure 3C**). This result indicates that SopE-mediated re-modelling of the F-actin cytoskeleton also affects other F-actin-dependent structures such as TJ.

### STM induce simultaneous microvilli collapse and ruffle formation

Effacement of MV, membrane ruffling and reticular F-actin formation were observed as outcome of STM infection. However, endpoint observations only provide restricted information about the kinetics of F-actin remodeling. Moreover, it remains open if STM induces membrane ruffling and MV collapse simultaneously, or if MV effacement is prerequisite for subsequent membrane ruffling.

**Figure 4:**
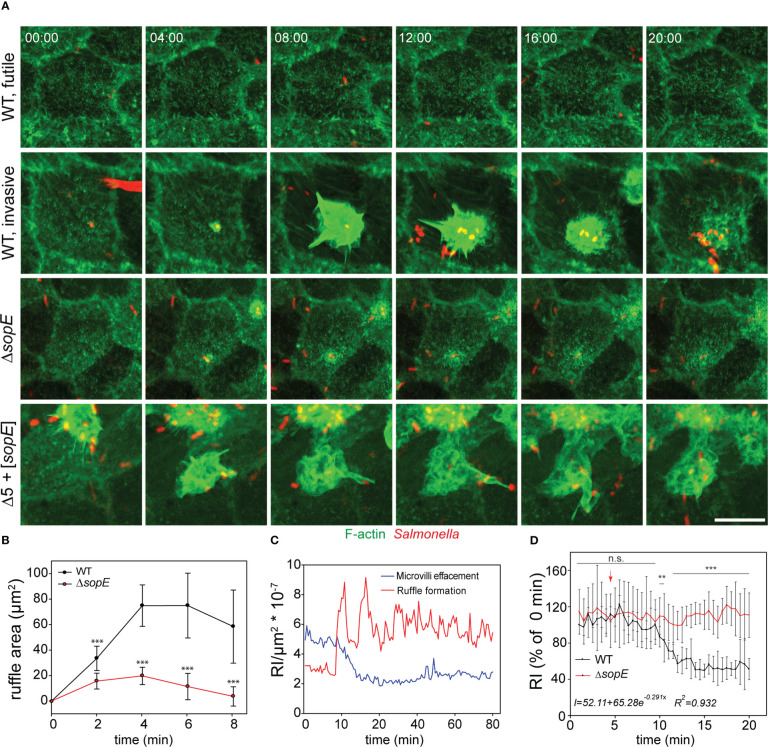
*Salmonella* infection of polarized cells induces simultaneous MV effacement and ruffle formation. **(A)** MDCK cells expressing Lifeact-eGFP were grown on fluorodishes for 5 d. Cells were infected with various strains as indicated and image acquisition by SDM at maximal acquisition speed (2-3 frames per min) over 120 min was started immediately after infection. MIP images are shown. Scale bar, 15 µm. Time stamp, min:sec. Cells were infected with STM WT, Δ*sopE* or Δ5 + [*sopE*] strains and events without entry of bacteria (WT, futile) or invasion (WT, invasive) are shown in time-lapse sequences **(A)**, **Movie 1**). **(B)** Reduced ruffles are formed by infection with the Δ*sopE* strain. Kinetics of changes in ruffle areas were estimated as described in **Figure 2C**. **(C)** MV collapse and ruffle formation occurs simultaneously. F-actin signals in two rectangular areas of the same infected cell were quantified. One area was set on a ruffle and the second area distant to the ruffle in an area maintaining MV. Quantification was performed from 10 min prior infection until 80 min after infection and the relative intensity (RI) of the Lifeact signal per µm^2^ was determined. **(D)** STM infection causes exponential decay of MV. Lifeact signals of MV-rich areas were quantified as in C) after infection with STM WT (black line) or Δ*sopE* (red line) strains. The arrow indicates the time point of STM docking to the host cell. Intensity was adjusted to 100% of the docking time point for 14 infection events per strain. The equation represents the behavior of signal decay in infected cells determined by fitting the measured signal to exponential decay model. Statistical analysis was performed as for **Figure 2** and is indicated as n.s., not significant; **P < 0.01; ***P < 0.001.

We performed live-cell imaging (LCI) of STM invasion of MDCK Lifeact-GFP cells with high temporal and spatial resolution using SDM (**Figure 4A**). Shortly after addition of the inoculum, WT bacteria reached the cell monolayer and adhered to cells. Only a subset of the adhesion events led to productive invasion processes, and another subset of WT bacteria did not induce any detectable alteration of the host cell actin (**Figure 4A**, ‘futile’). About 90-120 s after adhesion, individual bacteria triggered ruffle formation (**Figure 4A**, ‘invasive’).

The Lifeact-eGFP signal is directly proportional to amount of F-actin, thus can indicate MV collapse or effacement induced by STM. No alterations of MV were detected if WT adhesion did not result in ruffle induction, or if cells were invaded by single bacteria of STM Δ*sopE*. We observed effacement of the entire brush border for cells invaded by WT or Δ5 + [*sopE*]. For quantification of MV effacement, maximum intensity projection images from the entire cell infected by STM were analyzed in order to avoid missing signals due to Z-drift. We quantified lifetime and size of ruffles induced by WT and Δ*sopE* strains. Individual invasion events were located and ruffle dimensions were quantification from initiation to termination over ca. 45 min. This allowed definition of maximal ruffles sizes for individual invasion events. Analysis of the kinetics of invasion revealed radial extension of ruffles, initiating from the point of bacterial adhesion over an interval of 8 min. (**Figure 4B**). Although the duration of ruffles induced by both strains was the same, the area of ruffles induced by WT strain was larger than those triggered by Δ*sopE* strain. (**Figure 4B**). Time-lapse series revealed that the loss of MV occurred in parallel to the ruffle induction (**Figures 4A, C**, **Movie 1**). MV effacement is a consequence of the F-actin depolymerization, which might be active or passive. To address this question, we quantified the signal decay from those zones rich in MV during infection. This quantification demonstrated that the depolymerization of F-actin occurred exponentially (**Figure 4D**, insert), suggesting an active depolymerization process.

Further observations from LCI showed that the brush border architecture was not restored after ruffle retraction, instead reticular F-actin appeared 30 s before complete retraction of the ruffle and remained present over the entire imaging period (**Figure 4A** and **Movie 1**). F-actin depolymerization during ruffle formation was restricted to the apical side of infected cells (**Figure 4C**), since the basolateral F-actin cytoskeleton remained unaltered throughout the entire acquisition period (**Figure S3C**).

Infection with STM Δ5 induced no detectable alterations of the actin cytoskeleton (see **Figure 2E**), whereas infection with STM Δ5 + [*sopE*] resulted in extensive ruffle formation (**Figure 4A**). Although the membrane ruffling was similar for STM Δ5 + [*sopE*] and WT, the invasion by Δ5 + [*sopE*] often resulted in extensive spikes decorating the ruffle and F-actin protrusions (**Figure 4A**). Furthermore, cells invaded by STM Δ5 + [*sopE*] showed effacement of the entire brush border, while no alteration of MV was detected if cells were invaded by single bacteria of the Δ*sopE* strain (**Figure 4A**). In contrast to the effacement caused by STM Δ5 + [*sopE*] and WT STM, cells infected by the Δ*sopE* strain did not show any appreciable decay of F-actin signal from MV (**Figure 4D**).

Our results demonstrate that MV effacement and ruffle formation were simultaneously caused by STM during SPI1-T3SS-mediated invasion. In addition, MV were not restored after complete engulfment of STM, but reticular F-actin was newly polymerized. This also suggests that the increased F-actin polymerization at the ruffle led to consumption of the G-actin pool. Once this pool is exhausted, MV retract by enhanced depolymerization, as suggested by the exponential decay of the F-actin signal. The reduced F-actin polymerization activity observed during infection by the Δ*sopE* strain would not require consumption of the G-actin pool. In turn, MV integrity is not affected.

### Consumption of cytosolic G-actin during STM invasion results in microvilli collapse

**Figure 5:**
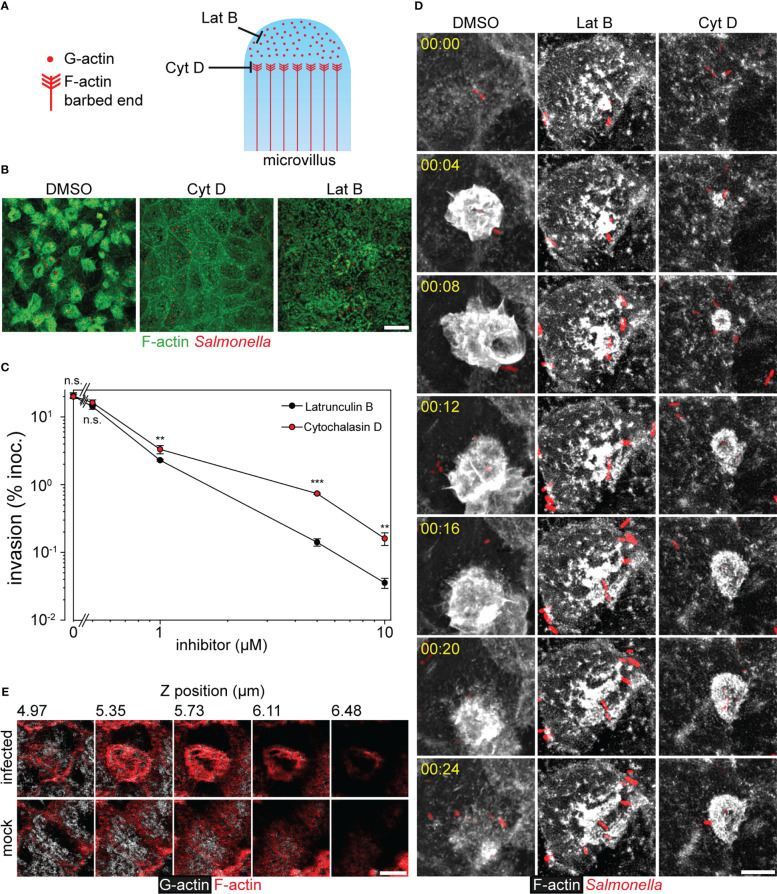
Consumption of the G-actin pool during invasion of *Salmonella* causes MV collapse. **(A)** Model for inhibitor action on the MV cytoskeleton. Latrunculin B (Lat B) sequesters G-actin monomers avoiding F-actin polymerization, while Cytochalasin D (Cyt D) binds to barbed ends impeding the incorporation of G-actin monomers to F-actin. Cyt D also blocks the depolymerization at the pointed ends of F-actin. **(B, C, D)** Effect of Lat B and Cyt D on the invasion of PEC. Infection of MDCK cells by STM WT was performed as in **Figure 1**, and solvent DMSO alone, Lat B or Cyt D were added to 10 µM final. **(B)** Representative micrographs are shown (F-actin, green; STM, red). **(C)** Invasion of STM WT was determined as in **Figure 2A** in presence of DMSO or various concentrations of Lat B and Cyt D added simultaneously to the inoculum (n = 3). **(D)** STM-induced membrane ruffling is not ablated, but decelerated in the presence of Cyt **(D)** Infection of MDCK Lifeact-eGFP cells was performed as described for **Figure 4** in the presence of 1 µM Lat B, 1 µM Cyt D or the same amount of DMSO. Still series of LCI are shown (Lifeact-eGFP, grey scale; STM, red; time stamp, h:min), corresponding to **Movie 2**. **(E)** Consumption of cytoplasmic G-actin at the apical side is caused by STM invasion. MDCK cells were infected with STM WT or mock infected for 25 min. After fixation and permeabilization, G-actin was stained with DNase I-Alexa488 (grey scale) and F-actin with actin stain 555 (red). Samples were analyzed by CLSM as before **(Figure 1A)** and micrographs show various positions of a Z-stack as indicated. Scale bars: 15 µm **(A)**, 10 µm **(C)**, 5 µm **(E)**. Statistical analysis was performed as for **Figure 2** and is indicated as n.s., not significant; **P < 0.01; ***P < 0.001.

To further test if membrane ruffling triggered by STM consumes the cytoplasmic G-actin and finally concludes in MV depolymerization, two inhibitors of F-actin polymerization were applied to invasion and LCI analyses. Latrunculin B (Lat B) binds G-actin and prevents polymerization, whereas Cytochalasin D (Cyt D) blocks F-actin polymerization by binding to the barbed ends of F-actin (Pollard and Mooseker, 1981), avoids F-actin depolymerization (Morris and Tannenbaum, 1980), and the interaction with cofilin at the pointed ends (Shoji et al., 2012) (**Figure 5A**). At concentrations of 10 µM, both inhibitors blocked the invasion by interrupting ruffle formation (**Figure 5B**). However, Lat B inhibited invasion more efficient than Cyt D (**Figure 5C**), since Lat B sequesters G-actin. Titration experiments showed that at inhibitor concentrations of 1 µM, invasion of Lat B-inhibited cells was 1.44-fold lower than of Cyt D-inhibited cells. Time-lapse series of WT infection in presence of 1 µM of either inhibitor supported this observation (**Movie 2**). Lat B-treated cells showed full disruption of F-actin cytoskeleton, and aggregation of actin at the apical side. However, ruffles were not fully formed by STM-triggered F-actin polymerization. In contrast, Cyt D-treated cells maintained MV and membrane ruffling proceeded slower and remained for more than 60 min without retraction. In DMSO-treated controls, the ruffle formation terminated in less than 20 min (**Figure 5D**). The distinct effects of the inhibitors indicate that STM*-*induced membrane ruffle formation deploys G-actin from sources other than F-actin in MV. If depolymerization of these structures is blocked by Cyt D, ruffle formation is dependent on the availability of the limited pool of cytoplasmic G-actin.

To follow the fate of the G-actin pool during STM infection, staining was performed with Phalloidin or DNase I to label F-actin or G-actin, respectively. At 25 min. p.i. G-actin in STM-infected cells was weakly stained by DNase I (**Figure 5E**). Most of the G-actin signal localized at ruffles, the origin of ruffles, and basolateral sides, but no G-actin was observed at the apical side. In contrast, non-infected cells presented homogenous distribution of G-actin with the exception of the nucleus. Taken together, the data support that membrane ruffles initiate by consumption of G-actin from the cytoplasmic pool. Moreover, these results show that only the apical F-actin cytoskeleton was affected by STM infection (**Figures 4**, **S3**).

### Src and MAPK pathways are not required for STM invasion of polarized epithelial cells

We set out to identify regulators of F-actin required for the STM-induced cytoskeletal remodeling that could explain MV effacement during STM invasion. In accordance with previous reports, we observed that cofilin, IQGAP1 and cortactin were recruited to ruffles triggered during invasion of MDCK cells by STM (**Figure S4**). Since all of these proteins are controlled by MAPK and Src kinases, we investigated the effect of pharmacological inhibitors PP1 (for Src kinase) and PD98059 (for ERK1/2 kinase) on STM invasion. Presence of 150 µM PD98059 or 50µM PP1 only mildly diminished invasion compared to cells treated with solvent DMSO (**Figure S4B, C**). These results indicate that although proteins involved in the Src and ERK1/2 kinase pathways are recruited to the ruffle formation in PEC, their function is secondary.

### Class I and class II myosins contribute to ruffle formation by STM during invasion of PEC

**Figure 6:**
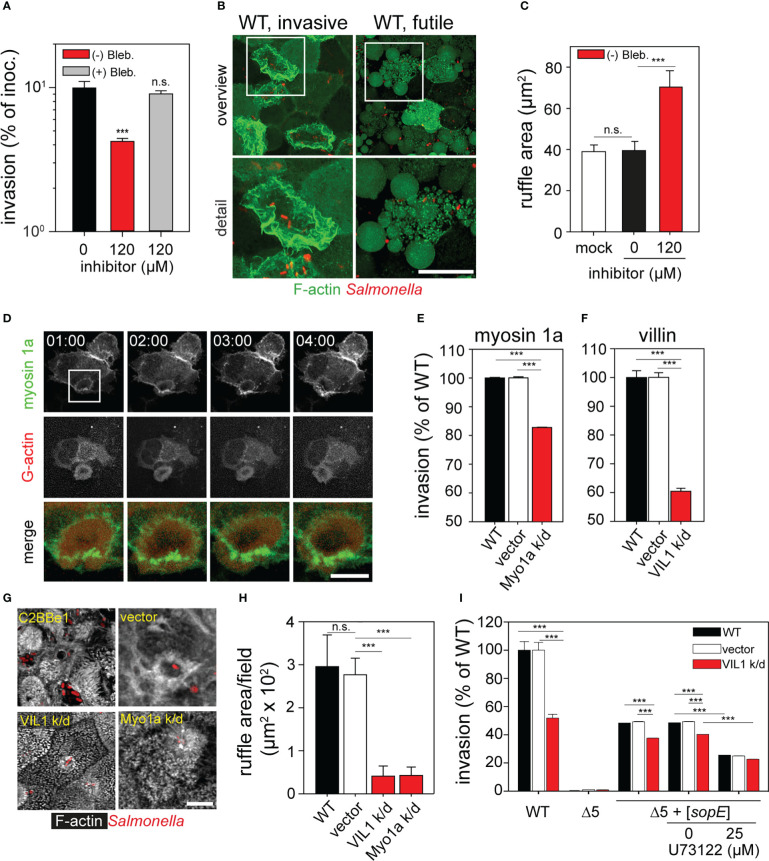
Role of class I and class II myosins and villin during invasion of polarized epithelial cells by *Salmonella*. **(A)** Inhibition of class II myosins impedes invasion. Infection of MDCK cells was performed as for **Figure 2A** in presence or absence of 120 µM (-) blebbistatin or its inactive enantiomer (+) blebbistatin. **(B)** Inhibition of class II myosins by S (-) blebbistatin inhibits the ruffle retraction and induced vesicle formation at the apical side of the MDCK cells. Representative infected cells with active invasion (WT, invasive) or without invasion (WT, futile) are shown. White boxes indicate areas shown in enlarged micrographs below. MDCK Lifeact-eGFP cells were infected with STM WT for 1 h with simultaneous addition of 120 µM (-) blebbistatin. **(C)** Size of ruffles triggered by STM after 25 min p.i. is enlarged as consequence of the treatment with S (-) blebbistatin as in **(A)**. Ruffle areas were quantified as in **Figure 2C**. **(D)** Myosin 1a is recruited to ruffles formed during STM invasion. MDCK cells were transfected with myosin 1a-eGFP (green) and β−actin-RFP (red) and infected with STM WT (not visible) as for **Figure 4A**. Images were acquired by SDM at the indicated time points (time stamp, min:sec) and the time-lapse series is shown in **Movie 3**. The white box indicates the section shown enlarged and merged. C2BBe1 cells were permanently transfected by empty vector or constructs expressing shRNA for knockdown of myosin 1a (Myo1a k/d) or villin (VIL1 k/d). Invasion assays with STM WT **(E, F, I)** or Δ5 + [*sopE*] **(I)** strains and microscopy analysis **(G, H)** were performed. **(E, F)**. Parental C2BBe1 or knockdown cells were infected with STM WT at MOI 10. Invasion was determined as before (**Figure 2**) and is expressed as percentage of invasion of the parental cell line. **(G, H)**. Silencing of villin and myosin 1a affects ruffle formation and MV effacement. **(G)** Micrographs of C2BBe1 cells were prepared as described for **Figure 1**. **(H)** Total ruffle areas were quantified as for **Figure 2C**. **(I)** Inhibition of PLCγ negatively affects invasion. C2BBe1 parental and knockdown cells were prepared as for **Figure S4**. C2BBe1 monolayers were treated with PLCγ inhibitor U73122 for 30 min prior infection. Results are expressed as percentage invasion of DMSO-treated cells. Scale bars, 20 µm (**B**, overview), 10 µm (**B**, detail, **D**, overview), 2.5 µm (**D**, detail, merge), 5 µm **(G)**. Statistical analysis was performed as for **Figure 2** and is indicated as n.s., not significant; ***P < 0.001.

Class II myosins are involved in the invasion of STM (Hanisch et al., 2011), thus invasion experiments in presence of class II myosin inhibitor (-) blebbistatin were performed. In contrast to previously published data (Hanisch et al., 2010), invasion of STM was affected by S (-) blebbistatin, since we observed a 3-fold reduction in invasion of MDCK cells (**Figure 6A**). Furthermore, STM infection of cells treated with S (-) blebbistatin led to induction of very large ruffles at 1 h p.i. (**Figures 6B, C** **)**. Quantification of ruffle areas demonstrated a two-fold increment in ruffles size for S (-) blebbistatin-treated cells (**Figure 6C**).

Interestingly, during LCI membrane vesicle formation at the apical side of cells treated with blebbistatin was observed (**Figure 6B**). The formation of vesicles at the apical side of the cells recalled the function of myosin 1a. Myosin 1a controls the tension in MV and formation of vesicles at the brush border, containing EPS8 L3, Ezrin, and sugar transporters (Mcconnell et al., 2009; Nambiar et al., 2009). Cells transfected with myosin 1a-eGFP were used to test the involvement of myosin 1a in invasion by STM. We observed the recruitment of myosin 1a-eGFP to ridges of ruffles triggered by STM, whereas β-actin-RFP was recruited to central areas of ruffles (**Figure 6D** and **Movie 3**). To test if myosin 1a actually contributes to STM invasion, C2BBe1 cells were stably transfected by a lentiviral construct encoding shRNA for knockdown (k/d) of myosin 1a (Myo1a k/d). In accordance to previous results (Tyska et al., 2005), the apical side of Myo1a k/o cells released large amounts of vesicles (**Figure S4D**). The invasiveness of STM in Myo1a k/d cells was reduced to 82.8 ± 0.1% (**Figure 6E**). In spite of the reduced effect of the knockdown of myosin 1a, ruffle size was also altered in absence of myosin 1a and MV, although with vesicles, were still present in cells infected by STM (**Figures 6G, H** **)**. These results demonstrate that class I and class II myosins contribute to the correct formation of ruffles triggered by STM. Abrogation of the function of these myosins impedes STM entry into host cells.

### Villin and PLCγ are required for STM invasion in polarized epithelial cells

The exponential decay of the F-actin observed during MV effacement indicates an active depolymerization process. Since contribution of cofilin is unlikely due to its absence in the normal brush border architecture (Ashworth et al., 2004), we investigated further candidates. Villin regulates the F-actin polymerization in MV by severing and capping F-actin, concluding in G-actin release (Bretscher and Weber, 1980; Ferrary et al., 1999; Ubelmann et al., 2013). Thus, the functions of villin may also be required for invasion of polarized cells by STM. We generated C2BBe1 cells with lentiviral transfection of shRNA for villin k/d (VIL1 k/d). These cells showed a normal distribution and structure of MV (**Figure 6G**, **Figure S4D**) similar to previous reports (Ferrary et al., 1999; Revenu et al., 2012). Invasion of VIL1 k/d cells was reduced to 58.7 ± 1.2% compared to parental C2BBe1 cells (**Figure 6F**). Further examination demonstrated that the size of STM-induced ruffles was highly decreased in contrast to those observed in parental cells. Membrane ruffles did not show large extensions as observed for infection with WT (**Figures 6G, H** **)**. In VIL1 k/d cells, MV were intact and invading bacteria surrounded by small ruffles, similar to the invasion phenotype of the Δ*sopE* strain (see **Figure 2**). These results show that MV are only effaced in presence of villin, which is probably associated with its severing activity.

Our results indicate that SopE dominantly controls the F-actin polymerization in host cells (**Figure 2**, **Figure 4**). Furthermore, we observed that F-actin depolymerization in MV is exponential and related to villin. However, the connection between SopE and villin is in this case unknown. Previous data already demonstrated that isoforms of phospholipase C-γ (PLCγ), PLCβ and PLA interact with Rac1 (Peppelenbosch et al., 1995; Athman et al., 2003; Jezyk et al., 2006). Thus, we inhibited PLCγ with pharmacological agent U73122, and determined the effect on invasion of the Δ5 + [*sopE*] strain. Invasion rates after treatment with U73122 of C2BBe1 and VIL1 k/d cells were 25% lower compared to that of the WT strain (**Figure 6I**). But in VIL1 k/d cells inhibited with U73122, no further inhibition of the STM invasion was detected. Therefore, SopE requires the function of PLCγ. If PLCγ is not activated, probably villin is not active. Similarly, if villin is absent, inhibition of PLCγ is futile, since there is not binding partner for PLCγ. Once villin is activated, depolymerization of F-actin culminates in MV collapse.

## Discussion

**Figure 7:**
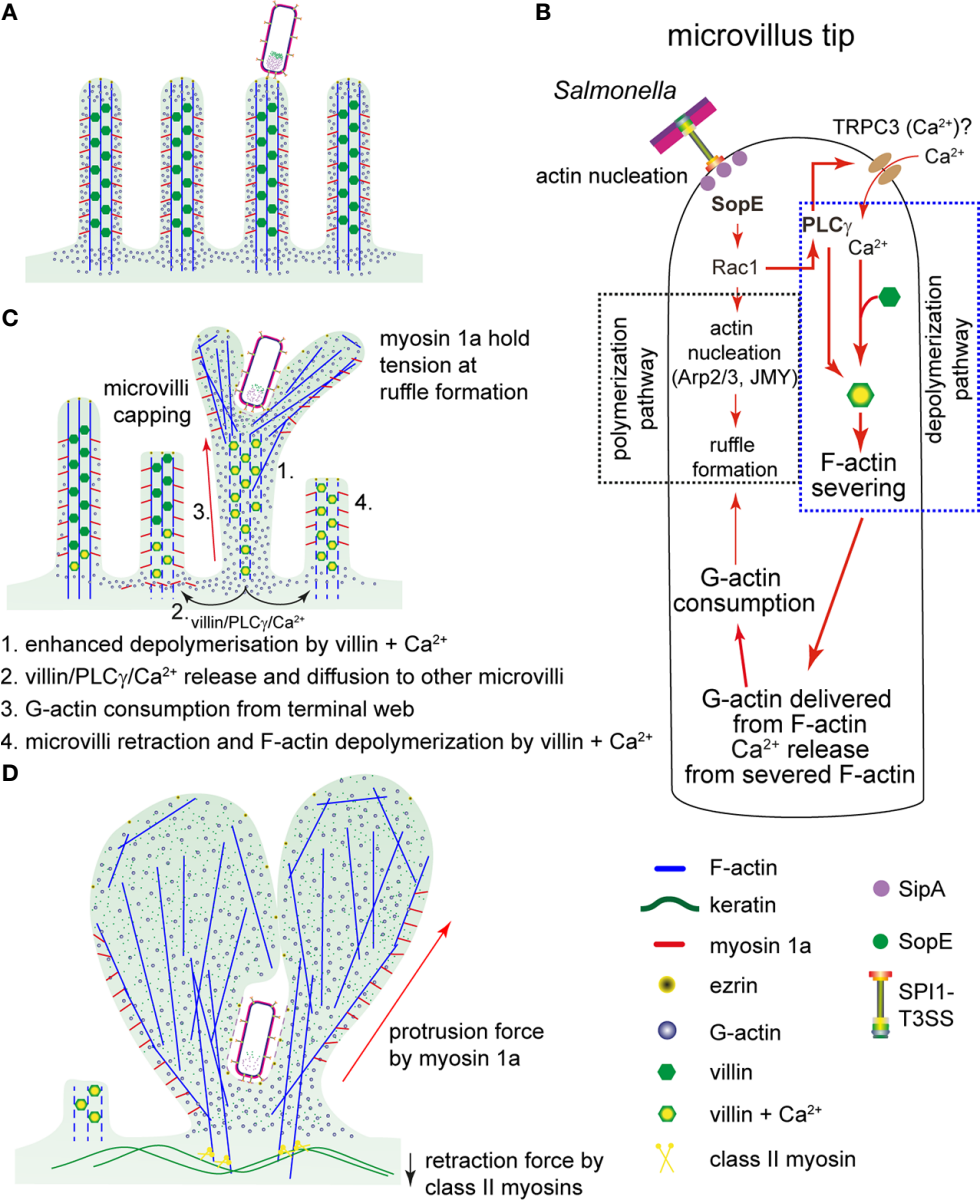
Model for a proposed sequence of manipulations of host cell actin by *Salmonella*. **(A)** After adhesion of STM to MV, the SPI1-T3SS translocates effector proteins. **(B)** SopE activates Rac1. Rac1 has a dual function in the invasion process of STM: i) Rac1 triggers the F-actin polymerization and ruffle formation by binding to F-actin regulators WASP/WASH and the activation of Arp2/3. ii) PLCγ, also activated by Rac1, activates villin that severs F-actin of MV. Thereby, G-actin and Ca^2+^ is released. The activation of PLCγ may also induce opening of Ca^2+^-specific channels, such as TRPC3. Resulting Ca^2+^ fluxes may contribute to the F-actin severing activity of villin. **(C)** Once F-actin in MV at the invasion point is depolymerized, Ca^2+^ and PLCγ-villin complexes would diffuse to the neighboring MV, again activating villin and consequently, F-actin depolymerization. Simultaneously, G-actin from the terminal web and cytoplasm is consumed by polymerization of F-actin in ruffles initiated by SopE. Since the concentration of G-actin becomes lower, other MV are capped and begin to depolymerize by the severing activity of villin (proposed sequence indicated by numbers). **(D)** The active depolymerization by PLCγ-villin complexes and consumption of G-actin conclude with MV collapse as observed in **Figure 4A** and measured in **Figure 4D**. Since ruffles triggered by STM are structures with constant F-actin polymerization *per se*, myosin 1a provides the protrusion force necessary to maintain the growth of the ruffle during the invasion process. Finally, cells lose the brush border and ruffles are completely formed.

Microvilli effacement has been previously observed during STM invasion of intestinal epithelium (Takeuchi, 1967), or in PEC models (Finlay et al., 1988; Gerlach et al., 2008), but the underlying molecular mechanisms have not been described. Here, we report the detailed analysis of the events and propose a new model for manipulation of the brush border by STM (**Figure 7**).

In the genetic background of an STM strain deleted of SPI1-T3SS effector proteins SipA, SopA, SopB, SopE and SopE2, expression and translocation of SopE only is sufficient to induce MV effacement and reorganization of F-actin in PEC. The effects of SopE interaction with Rac1 enable STM to coordinate its internalization into polarized cells, which requires both destruction of brush border barrier functions and induction of membrane ruffles. So far, the only targets described for SopE are Rac1 and Cdc42 (Hardt et al., 1998a; Friebel et al., 2001). Therefore, all cellular events observed during STM invasion exclusively depend on signaling triggered by SopE-Rac1. This includes recruitment of WASP/WAVE2 and Arp2/3, and stimulation of PLA and PLCγ activation (Peppelenbosch et al., 1995; Shi et al., 2005; Jezyk et al., 2006; Hanisch et al., 2010). Whereas WASP-WAVE2-Arp2/3 trigger F-actin polymerization, PLCγ directly acts on Ca^2+^ uptake (Wen et al., 2006) and villin activation (Athman et al., 2003) that lead to F-actin depolymerization.

Results presented here also demonstrate that inhibition of PLCγ and knockdown of villin limited the invasion of STM. These findings are in line with work of Lhocine et al. (2015) that reported the requirement of villin for STM apical invasion, based on reduced STM invasion of villin k/d cells, and reduced STM-mediated intestinal pathology in villin-deficient mice. Previous published data showed the activation of PLA by Rac1 induces leukotrienes responsible for Ca^2+^ uptake during STM invasion in Henle-407 cells (Pace et al., 1993). In addition, the increase of [Ca^2+^] in brush borders primes the F-actin depolymerization by activation of the severing activity of villin (Glenney et al., 1981; Ferrary et al., 1999), which is reinforced by the binding of PLCγ to villin (Athman et al., 2003; Revenu et al., 2004). Since our data showed no MV destruction in sh VIL1 k/d C2BBe1 cells after STM infection, we suggest that villin after stimulation of PLCγ by Rac1-SopE is responsible to sever F-actin in MV (**Figure 7B**). Furthermore, the reduced invasion in PLCγ-inhibited cells may considerably limit the activation of villin reducing the F-actin depolymerization.

Since membrane ruffles triggered by SopE radially grew over the apical side of the cells, we propose that F-actin remodeling would also radially deliver complexes of villin-PLCγ that may activate depolymerization in other regions of the apical side of the host cells (**Figure 7C**) causing MV effacement. In contrast, if ruffle expansion does not occur due to the absence of SopE, then villin-PLCγ cannot propagate to other regions of the cell.

SopE translocation also is dominant in destruction of epithelial barrier function and tight junction integrity. These effects can be explained by the recruitment of Rac1 to ruffles at the apical side and consumption of G-actin. Since both Rac1 and G-actin are required to maintain TJ integrity, recruitment to ruffles at the apical side affects integrity of lateral cell-cell connections. The depolymerization mechanism delivers more G-actin necessary to sustain the formation of ruffles by STM. We showed that STM infection still triggered ruffles in cells inhibited by Cyt D, but these ruffles grew slowly and no MV effacement occurred. This indicates that STM-induced membrane ruffling employs different sources of G-actin: the cytoplasmic pool of G-actin is recruited first, followed by G-actin from F-actin structures such as MV. Recent work showed that migration of PEC in the intestine and in cell culture models requires the severing activity of villin. Cells lacking villin or its severing domain migrated slower than WT cells, and lamellipodia formation was highly diminished (Ubelmann et al., 2013). Furthermore, actin found first in MV was later detected in lamellipodia, which might probably be due to the reduced ratio F-/G-actin of 7:3 observed in PEC (Stidwill and Burgess, 1986). Thus, in analogy to this system and in view of the low amount of G-actin in PEC, SopE-induced F-actin polymerization quickly consumes the cytoplasmic G-actin. Thereafter, depolymerization of other F-actin structures such as MV would provide more actin monomers, allowing the continuation of F-actin polymerization in ruffles (**Figure 7C**).

In addition to villin, ADF/cofilin could also contribute to F-actin depolymerization. Nevertheless, activation of Rac1 by SopE induces the phosphorylation of the LIM kinase responsible for the inactivation of cofilin. F-actin is also protected against cofilin by decoration of SipA, as demonstrated *in vitro* (Mcghie et al., 2004). Proteome analyses of the murine intestinal brush border did not indicate presence of this depolymerization factor (Mcconnell et al., 2011). Hence, cofilin is unlikely to be the factor responsible for MV effacement.

Further molecular analysis of MV effacement showed that class I and II myosins contribute to the ruffle structure. In contrast to results of Hanisch et al. (2011), MDCK cells inhibited by S (-) blebbistatin were less efficiently invaded by STM. Blebbistatin-treated cells revealed extremely enlarged ruffle morphology. Class II myosins are found at the terminal web of MV and they mislocalize in absence of plastin I (Grimm-Gunter et al., 2009). Since class II myosins are responsible for F-actin retraction, their inhibition by blebbistatin would therefore interfere the ruffle retraction at the terminal web. Then, these ruffles remain open and STM internalization fails.

The large size of ruffles observed was attributed to the protrusion force of myosin 1a. After k/d of myosin 1a expression, cells infected by STM only showed small ruffles, similar to those observed in shVIL1 k/d cells. Myosin 1a keeps the tension of MV and regulates the formation of vesicles (Tyska et al., 2005; Mcconnell and Tyska, 2007; Mcconnell et al., 2009; Nambiar et al., 2009). Absence of myosin 1a during ruffle formation could abrogate the protrusion force necessary during the F-actin polymerization. Therefore, myosin 1a could play a role by providing the tension force for membrane ruffling. This, in turn, would permit efficient ingress of STM into host cells. Subsequent to MV effacement and invasion, we observed the transient formation of reticular actin on the apical side of PEC. The reticular actin may represent futile reformation of MV, for example due to loss of actin binding proteins EPS8 and IRTKS, improper branching and separation of growing F-actin filaments (Gaeta et al., 2021).

A recent study investigated the interaction of STM with the murine cecal epithelium, and the results challenge previous models for STM invasion (Fattinger et al., 2020). Analyses of STM entry events indicated preferential interactions at cell-cell junctions, and enterocytes next to goblet cells, only limited apical membrane remodeling referred to as discreet entry structures, and a primary role of SipA in invasion, rather than of SopE, SopE2 and SopB (Fattinger et al., 2020). Cell culture models such the PEC models applied in our work are limited in representation of the complex cellular organization of the intestinal epithelium, and lack factors such as mucus and intestinal microbiota. Yet, *in vivo* analyses remain limited in capturing dynamic events such as trigger invasion by LCI, and transient alterations of MV architecture thus may be missed. We consider intestinal organoids as attractive infection model that resemble key histological features of the intestinal epithelium, allow LCI of infection, as well as analyses of infection of human-derived material (Puschhof et al., 2021).

In summary, our data demonstrate how STM remodels the F-actin cytoskeleton in PEC and reveal molecular mechanisms leading to MV effacement. Our model suggests that SopE-Rac1 interaction has dual functions in remodeling of the F-actin cytoskeleton. While F-actin polymerization is induced by SopE-Rac1, WASP and Arp2/3, activation of PLCγ and villin by SopE-Rac1 contributes to depolymerize F-actin structures as a mechanism that may ensure a new source of G-actin. We also identified that myosin 1a and class II myosins are necessary in signaling and reorganization of F-actin during the ruffle formation and retraction in PEC. Therefore, the translocation of SopE into host cells is essential to trigger both mechanisms, and provides the substrates for ruffle formation during STM invasion. Changes induced by SopE-Rac interaction may contribute to the recruitment of other proteins targeted by SopB, SipA and SopE2. Hence, the functions of effector proteins are cooperative, rather than redundant, as deduced from analyses in non-polarized cell models. This conclusion calls for further detailed studies of the interaction of these effectors with microvillar proteins in PEC. Such work should provide insights into novel mechanisms involved in the host cell signaling to response against the infection by STM, and so far unknown physiological consequences of the infection by this intestinal pathogen.

## Materials and methods

### Bacterial strains, construction of mutants and plasmids for complementation

*Salmonella enterica* sv. Typhimurium strain SL1344 was used as wild-type (WT) strain and mutant strains were isogenic to either WT. Bacterial strains used in this study are listed in **Table 1**. In order to obtain mutant strains of *sopA*, *sopB* and *sopE2* in the strain background of SL1344, gene replacements by an *aph* cassette were generated in NCTC 12023 by Red-mediated recombination (Datsenko and Wanner, 2000) using pKD13 as template and primers specified in **Table S1**. Subsequently, P22 transduction according to standard methods (Maloy et al., 1996) was used to move the mutations to SL1344. Plasmids used for complementation of mutations, or expression of fluorescent proteins are listed in **Table 2** and construction is described in Suppl. Materials.

**Table 1:** Bacterial strains used in this study.

Designation	Relevant characteristics	Reference
*S. enterica* serovar Typhimurium SL1344 strains		
SL1344	wild type, Sm^R^	lab stock
M712 (or Δ5)	Δ*sipA sopA sopB sopE sopE2*	(Ehrbar et al., 2004)
MvP1450	*sopB*::*aph*, Km^R^	this study
MvP1459	*sopE2*::*aph*, Km^R^	this study
MvP1473	*sopA*::*aph*, Km^R^	this study
SB161	*invG*	(Kaniga et al., 1994)
SB225	*sipA*::*aph*T, Km^R^	(Kaniga et al., 1995)
SB856	*sopE*::*aph*T, Km^R^	(Hardt et al., 1998a)

**Table 2:** Plasmids used in this study.

Designation	Relevant characteristics	Reference
Complementation		
pWSK29	low copy number, Amp^R^	(Wang and Kushner, 1991)
p4041	pWSK29 P*_sopA_*::*sopA*::HA	this study
p4042	pWSK29 P*_sopB_*::*sopB*::HA	this study
p4043	pWSK29 P*_sopE_*::*sopE*::HA	this study
p4044	pWSK29 P*_sopE2_*::*sopE2*::HA	this study
p4040	pWSK29 P*_sicA_::sipA*::HA	this study
Mutagenesis		
pKD4	Red deletion template, *aph*	(Datsenko and Wanner, 2000)
pKD13	Red deletion template, *aph*	(Datsenko and Wanner, 2000)
pKD46	Red recombinase, Amp^R^	(Datsenko and Wanner, 2000)
pCP20	FLP recombinase, Amp^R^	(Datsenko and Wanner, 2000)
Fluorescent protein expression		
p3589	P*_rpsM_*::mCherry in pETcoco, Cm^R^	this work
pWRG439	P*_rpsM_*::mTagRFP in pFPV25.1, Amp^R^	Roman G. Gerlach
Transfection		
Lifeact-eGFP		(Riedl et al., 2010)
pRFP β-actin		Theresia Stradal
pMyo1A-eGFP		(Tyska and Mooseker, 2002)
pIQGAP1-eGFP		(Ren et al., 2005), (Addgene 30112)
pCortactin-RFP		(Taylor et al., 2011), (Addgene 27676)
pCofilin-RFP		(Taylor et al., 2011), (Addgene 27687)

### Cell lines and culture conditions

Cell lines were cultured at cells were incubated at 37°C in a humidified atmosphere containing 5% CO_2_. For invasion assays and microscopy analyses, MDCK clone Pf were used as standard cell culture model, kindly provided by the Nephrology department of the University Hospital Erlangen. Confluent monolayers in 25 cm^2^-cell culture flasks were seeded each week in a new 25 cm^2^ flask with MEM supplemented with 1 x non-essential amino acids (PAA, Germany), 10% inactivated fetal calf serum (FCS, Sigma, Germany) and 1 x Glutamax (PAA, Germany). Cell line C2BBe1, a derivate of CaCo2 cell line (ATCC CRL-2102) was used as human polarized epithelial cell line to analyses of STM interactions. These cells were cultured in DMEM high glucose without pyruvate (PAA, Germany), containing Glutamax, 10% FCS and 2.5 µg x ml^-1^ holo-transferrin (Sigma-Aldrich, Germany). For cultivation, media were supplemented with penicillin/streptomycin (PAA, Germany). Medium was changed every third day. Medium are replaced by antibiotic-free medium at least one day prior infection. Cells were seeded at 10^5^ cells per 12 mm polycarbonate filter insert (0.4 µm pore size, Millipore, Germany). The trans-epithelial electrical resistance (TEER) was measured every third day with a platinum electrode and an Ohmmeter EVOM (World Precision Instruments, USA). Cells were cultured until a TEER of 500-700 Ω per well was observed, usually for 10 to 15 d. TEER was determined prior and after infection by STM as indicator to epithelial barrier integrity. Generation of knock-down cell lines is described in Supplementary Materials and Methods.

### Invasion assays

Five days prior to infection, MDCK cells were seeded at 1 x 10^5^ cells per well in 24-well plates. At least 4 h before infection, the medium was substituted by medium without antibiotics. Bacterial strains were precultured in LB overnight at 37°C with continuous aeration in glass test tubes in a roller drum. Overnight cultures were diluted 1:31 in fresh LB and cultured for 3.5 h as above. Cultures were adjusted to OD_600_ = 0.2 in PBS and a master mix was prepared in MEM. Cells were infected in triplicates with each strain from the master mix at multiplicity of infection (MOI) of 5. After invasion for 25 min, non-internalized bacteria were removed by washing three times with PBS. Thereafter, fresh medium containing 100 µg x ml^-1^ gentamicin was added for 1 h. Finally, infected cells were washed five times with PBS and lysed with 0.5% deoxycholic acid for 10 min. Lysates were diluted and platted onto Mueller-Hinton agar (BD, Germany) plates with an Eddy Jet spiral platting instrument (IUL Instruments, Barcelona). Plates were incubated at 37°C overnight and numbers of colonies were counted.

For infection of monolayers in transwell filters, bacterial strains were added at MOI 50 and TEER was recorded each 15 min for the first hour, thereafter each 30 min until 2.5 h. Finally, cells were washed thrice with pre-warmed PBS and fixed with methanol at -20°C overnight.

### Immunostaining

For imaging, bacterial strains harboring p3589 for constitutive expression of mCherry were used. 1 x 10^5^ cells were seeded on cover slips as for invasion assays. Bacteria were diluted in MEM to an OD_600_ of 0.2 and applied for infection at MOI 50. Infections were made in duplicate at least three times independently. After 15, 25, or 60 min, the infection was stopped by washing cells with PBS four times. For fixation, 3% PFA in PBS was added and cells were incubated for 1 h at 37°C. After fixation or any subsequent incubation with reagents or antibodies, cells were washed thrice with PBS at 37°C. Fixed cells were permeabilized by incubation for 15 min at 37°C with 0.5% Triton X-100 (Sigma-Aldrich, Germany) in blocking solution consisting of 2% BSA (Biomol, Germany), 2.0% Goat serum (Gibco, Germany) in PBS in a humid chamber. Antibodies were diluted in blocking solution and incubations were performed at 37°C in a humid chamber. Rabbit anti Ezrin was diluted 1:500 and incubated with the samples for 1 h. Alexa488-conjugated Phalloidin (Invitrogen, Germany) was added in 1:200 dilution and incubated 45 min at 37°C. Cover glasses were then mounted on glass slides with Fluoroprep (Biomerieux, France), sealed with Entellan (Merck, Germany) and kept in the dark at 4°C. C2BBe1 cells were also used for microscopy analysis of tight junction integrity. After fixation with cold methanol overnight at -20°C, tight junctions were stained with rabbit anti ZO-1 at a dilution of 1:200 for 2 h. Alexa488-conjugated anti-rabbit secondary antibody was applied 1:500 for 1 h. All washing steps were performed as for MDCK cells. Finally, filter inserts were recovered and mounted between a cover slip and glass slide as described above.

### Microscopy analysis and live cell imaging

Fixed samples were observed with a SP5 II confocal laser-scanning microscope (CLSM, Leica Microsystems Wetzlar, Germany). Images were acquired using a 100x objective with a numerical aperture of 1.51 and 1 Airy unit for the pinhole, the pixel size of the images was 70.85 x 70.85 nm. The 488 nm argon laser line was used for Alexa488-conjugated antibodies and eGFP. The HeNe 543 nm laser line was used for excitation of Alexa568-conjugated antibodies and mCherry or mTagRFP. Images were acquired with Leica Acquisition Software V. 2.3.6 and further processed with Imaris V. 7.6.1 (Bitplane, Switzerland) and FIJI (Max-Planck Institute for Cell Biology, Dresden, Germany). LCI of MDCK cells expressing Lifeact-eGFP, Myo1a-eGFP, β-actin-RFP, IQGAP1-eGFP, Cortactin-mCherry or cofilin-RFP and mCherry-, GFP-, or mTagRFP-expressing STM strains was performed using a CellObserver microscopy system (Zeiss, Germany) equipped with Yokogawa spinning disc unit. Images were acquired for 120 min starting shortly after infection at maximal speed with intervals of 100 to 200 ms, recording Z-stacks with distances of 0.30 – 0.35 µm between Z-planes. A water immersion objective with a numerical aperture of 1.333 was used. Acquisition was performed with either a cooled CCD camera (CoolSNAP HQ^2^, Photometrics) with a chip of 1,040 x 1,392 pixels for high spatial resolution, or an EM-CCD camera (Evolve, Photometrics) with a chip of 512 x 512 pixels for high sensitivity. Acquisition and processing of time-lapse images was performed with AxioVision 4.8.2 or ZEN 2011 software (Zeiss). Images from CLSM or SDM were deconvolved with Huygens V.4.2 using a theoretical point spread function. Bleaching and Z-drift were also corrected with Huygens.

### Atomic force microscopy

AFM measurements were conducted using the NanoWizard II AFM system (JPK Instruments AG, Berlin, Germany). High-resolution surface images were acquired by operating the AFM under ambient conditions in soft contact mode using silicon nitride AFM probes with a nominal force constant of 0.06 N/m (SiNi, Budget Sensors, Wetzlar, Germany). Samples were prepared as described above. For each sample, topographic overview images with a 90 x 90 µm scan area were taken before zoom-ins were generated. All images were polynomial fitted and unsharpened mask filtered using JPK data processing software (JPK Instruments AG). 3D projections of height profiles are shown, tilted 12° in X direction.

To correlate surface structures recorded by AFM with the cytoskeleton, epifluorescence images of Lifeact-eGFP cells were acquired and aligned with AFM images by matching landmarks observed in both images using the transform tool in Adobe Photoshop.

## Data availability statement

The raw data supporting the conclusions of this article will be made available by the authors, without undue reservation.

## Author contributions

AF-L, NH and MH designed the research, analyzed the data, and wrote the manuscript. AF-L, CD and NH performed the research. All authors contributed to the article and approved the submitted version.

## Supplementary material

Supplementary Figure 1
*Salmonella*-induced F-actin reorganization of the apical side of polarized epithelial cells. Micrographs of Z-stacks for cells shown in **Figure 1** are displayed. F-actin signals for reticular F-actin, microvilli and ruffles were only observed at the apical side of host cells. Scale bar, 15 µm.

Supplementary Figure 2 SPI1-T3SS effector proteins have divergent effects on F-actin reorganization in polarized epithelial cells. Analyses of invasion of MDCK **(A, B)**, C2BBe1 **(C, D, G)**, or HeLa **(E, F, H)** cells were performed with various STM strains as described for MDCK. C2BBe1 were grown on transwell filter as indicated for TEER measurements. HeLa cells were seeded at 250,000 cells per well and infected the next day at MOI 50. Infection was performed as indicated with STM WT, *invG*, Δ*sipA*, Δ*sopA*, Δ*sopB*, Δ*sopE*, or Δ*sopE2* strains, or strain Δ5, without or with complementation plasmids for *sipA, sopA*, *sopB*, *sopE* or *sopE2*. Levels of invasion were compared to WT **(A, C, E)** or strain Δ5 **(D, F)**. Deletion of *sipA* and *sopB* reduced invasion **(A)** of MDCK cells, and affected morphology of membrane ruffles in MDCK cells **(B)**. Complementation of *sipA* in strain Δ5 induces actin recruitment around the bacterial cell **(B)**. Complementation of only *sopB* or *sopA* was not sufficient to induce changes in the F-actin cytoskeleton **(B)**. Deletion of *sopE* decreases invasion of C2BBe1 **(C)** and HeLa cells **(E)**. Complementation with *sopE* in strain Δ5 recovers invasion of C2BBe1 (D) and HeLa cells (F). B, G, H) Micrographs of infected cells were acquired by CLSM as described for **Figure 1**. Scale bar, 20 µm.

Supplementary Figure 3 MDCK cells transfected with Lifeact-eGFP allow observation of F-actin dynamics during *Salmonella* infection. **(A)** Microvilli dynamics at the apical side of MDCK cell. MDCK Lifeact-eGFP cells were seeded as described in **Figure 2A** and LCI was performed by SDM. Images from the apical and basolateral sides are shown and **Movie 4** shows the time-lapse sequence for the apical side. **(B)** F-actin changes occur only at the apical, but not at the basolateral side of cells. The apical and basolateral sides of WT-infected cells from **Figure 4**, as well as of cells infected by Δ*sopE*, Δ5 + [*sopE*] strains are shown. Scale bars, 10 µm **(A)**, 15 µm **(B)**. Time stamp, min:sec.

Supplementary Figure 4 IQGAP1, cortactin and cofilin are involved in the ruffle formation, but signaling pathways ERK1/2 and Src are not necessary. **(A)** The actin scaffold proteins IQGAP1 and cortactin, as well as the depolymerizing factor cofilin are recruited to the ruffle triggered by STM. MDCK cells were permanently transfected as stated in Material and Methods with plasmids carrying IQGAP1-eGFP, cortactin-RFP or cofilin-mCherry. Cells were infected as for **Figure 4A**. Images were continuously acquired by SDM for 30 min and MIP images are shown. Signal intensities are represented by pseudo-color scale (fire), the maximal and minimal intensities are given in the respective time series (time stamp, min:sec). See **Movie 5** for time-lapse sequences. **(B, C)** ERK1/2 and Src kinases are not necessary for STM invasion of polarized cells. Inhibition experiments were carried out with the ERK1/2 kinase inhibitor PD98059 **(B)** and Src kinase inhibitor PP1 **(C)** or the solvent controls (DMSO). Inhibitors were added 30 min before infection and STM strains were used for infection as described for **Figure 2A**. Statistical analysis was performed comparing the levels of invasion of inhibited cells to DMSO-treated cells (negative control). **(D)** C2BBe1 cells were grown for 10 d and processed for AFM as described in **Figure 1B**. Scale bars, 4 µm **(A)**, and 2 µm **(D)**.

Supplementary Table 1 Oligonucleotides used in this study.

Movie 1 Lifeact MDCK cells infected with *Salmonella* WT, Δ*sopE* strain, and Δ5 strain complemented with *sopE*. Time-lapse series were recorded for 60 min. Time stamp, h:min:sec.ms. Scale bars, 5 µm. The movie corresponds to **Figure 4A**. Link to Movie: https://myshare.uni-osnabrueck.de/f/16cd4a9658124c6683eb/.

Movie 2 Effect of Lat B and Cyt D on invasion of polarized epithelial cells. Infection of MDCK Lifeact-eGFP cells was performed as described for in the presence of 1 µM Lat B, 1 µM Cyt D, or the same volume of DMSO. LCI was performed by SDM for registration of Lifeact-eGFP (grey scale) and STM (red) signals. Scale bars, 5 µm; time stamp, h:min. The movie corresponds to **Figure 5D**. Link to Movie: https://myshare.uni-osnabrueck.de/f/b0fcac35ab304e9ca93a/.

Movie 3 Myosin 1a is recruited to ruffles formed during *Salmonella* invasion. MDCK cells were transfected with myosin 1a-eGFP (green) and β−actin-RFP (red) and infected with STM WT (not visible) as for . Images were acquired by SDM at the indicated time points. Scale bar, 10 µm. The movie corresponds to **Figure 6C**. Link to Movie: https://myshare.uni-osnabrueck.de/f/e38977c13a0b4dceb25f/.

Movie 4 Observation of microvilli dynamics at the apical side of MDCK cell by Lifeact-eGFP. MDCK Lifeact-eGFP cells were seeded as described in and LCI was performed by SDM for a time-lapse sequence of F-actin dynamics at the apical side. Scale bars, 10 µm. Time stamp, min:sec. The movie corresponds to **Figure S3A**. Link to Movie: https://myshare.uni-osnabrueck.de/f/e38977c13a0b4dceb25f/.

Movie 5 IQGAP1, cortactin and cofilin are involved in ruffle formation. MDCK cells were permanently transfected for expression of IQGAP1-eGFP, cortactin-RFP or cofilin-mCherry. Images were continuously acquired by SDM for 30 min and MIP images are shown. Signal intensities are represented by pseudo-color scale (‘fire’), the maximal and minimal intensity are given in the respective time series (time stamp, min:sec). Scale bars, 4 µm. The movie corresponds to **Figure S4A**. Link to Movie: https://myshare.uni-osnabrueck.de/f/dfea6a215bfe4a278289/.
