# Supplementary material for: Manipulation of microvillar proteins during Salmonella enterica invasion results in brush border effacement and actin remodeling
Source: Front Cell Infect Microbiol. 2023 Mar 2;13:1137062. doi: 10.3389/fcimb.2023.1137062 (PMC10018140; doi:10.3389/fcimb.2023.1137062)
Supplement: Supplementary file 1 [file DataSheet_1.pdf]

Supplementary Materials to:

Manipulation of microvillar proteins during *Salmonella enterica* invasion results in brush border effacement and actin remodeling

Alfonso Felipe-López, Nicole Hansmeier, Claudia Danzer, Michael Hensel

Supplemental Materials and Methods

Generation of plasmids for complementation

For complementation of *sipA*, *sopA*, *sopB*, *sopE* or *sopE2* deletions, the corresponding genes were amplified from SL1344 genomic DNA introducing 3' HA tag sequences for detection of the encoded proteins by immunoblotting. The products were cloned in low copy number vector pWSK29 and *E. coli* DH5α was used to propagate plasmids. Since *sipA* is the terminal gene of the *sicA sipBCDA* operon, the P_{sicA} promoter was amplified and cloned upstream of *sipA*::HA to generate p4040. Mutant strains were transformed with complementation plasmids listed in Table S2.

To test integrity of effectors expressed by cloned genes, complemented strains were subcultured as for cell invasion. Bacteria were centrifuged at 13,000 x *g* at 4 °C for 30 min and the pellet was adjusted to OD_{600} 1.0 with SDS-PAGE sample buffer. Lysates were loaded into SDS-PAGE gels, proteins separated, and semi-dry blotting with Towbin buffer system was used to transfer protein onto 0.22 μm nitrocellulose membranes. Detection of tagged proteins was performed using high-affinity primary rat HA-antibody (Roche), horseradish peroxidase-conjugated goat secondary antibody and ECL detection. Clones expressing HA-tagged effectors of the anticipated molecular weight were used for further experiments.

Transfection of MDCK cells

MDCK cells were transfected with various plasmids listed in Table S2 by a modification of the CaPi method (Graham and van der Eb, 1973;Deen et al., 1997). Briefly, 5.0 x 10^5 cells were seeded in a Petri dish 24 h before transfection. 10 μg of plasmid DNA were solved in 63 μl of 2 M $CaCl_2$. To this solution, 500 μl of HGBS (280 mM NaCl, 10 mM KCl, 1.5 mM Na_2HPO_4, 12 mM dextrose, 50 mM HEPES; pH 7.05) were added slowly during strong stirring. DNA was

precipitated during incubation on ice for 20 min. 50 µl of this solution were dropped into the cell culture. Thereafter, transfected cells were incubated for 6 h at 37 °C at 5.0% CO_2. Finally, medium containing 100 µg x ml^{-1} geneticin (Gibco, Life Technologies, Germany) was added. Medium was changed daily until confluence of the cell layer was reached. At this stage clones were seeded at densities of 5.0 x 10^4 to 1 x 10^6 cells per 90 mm Petri dish, and geneticin was increased to 150 µg x ml^{-1}. Clone K4 showed 100% Lifeact-eGFP expressing cells, retained the typical morphology of MDCK cells and the infection rates were similar to the non-transfected cells (see Fig. S3). Thus, clone K4 was used for subsequent analyses. MDCK cells expressing Myo1a-eGFP and RFP-β-actin were enriched by FACS.

Generation of lentiviral particles

Primers for the amplification of the anti-sense sequence for myosin 1a (shMyo1a) and villin 1 (shVIL1) were designed with BLOCK-iT™ RNAi Designer (Life Technologies) and are listed in Table S1. To generate the loop sense anti-sense sequence, the sequence TTCAAGAGA between the sense and the anti-sense sequence was inserted by PCR, hence a shRNA can be expressed. These sense/anti-sense sequences were cut with MluI and ClaI, and inserted into the vector pLVTHM digested by MluI and ClaI. Lentiviral particles for transfection with shRNA constructs were prepared as previously described (Wiznerowicz and Trono, 2003). As negative control, the empty vector was used. Samples were scanned by AFM to control microvilli integrity after knockdown of target functions.

References:

Deen, P.M., Rijss, J.P., Mulders, S.M., Errington, R.J., Van Baal, J., and Van Os, C.H. (1997). Aquaporin-2 transfection of Madin-Darby canine kidney cells reconstitutes vasopressin-regulated transcellular osmotic water transport. *J Am Soc Nephrol* 8**,** 1493-1501.

Graham, F.L., and Van Der Eb, A.J. (1973). Transformation of rat cells by DNA of human adenovirus 5. *Virology* 54**,** 536-539.

Wiznerowicz, M., and Trono, D. (2003). Conditional suppression of cellular genes: lentivirus vector-mediated drug-inducible RNA interference. *J Virol* 77**,** 8957-8961.
