# Supplementary figures and images for: Manipulation of microvillar proteins during Salmonella enterica invasion results in brush border effacement and actin remodeling
Source: Front Cell Infect Microbiol. 2023 Mar 2;13:1137062. doi: 10.3389/fcimb.2023.1137062 (PMC10018140; doi:10.3389/fcimb.2023.1137062)

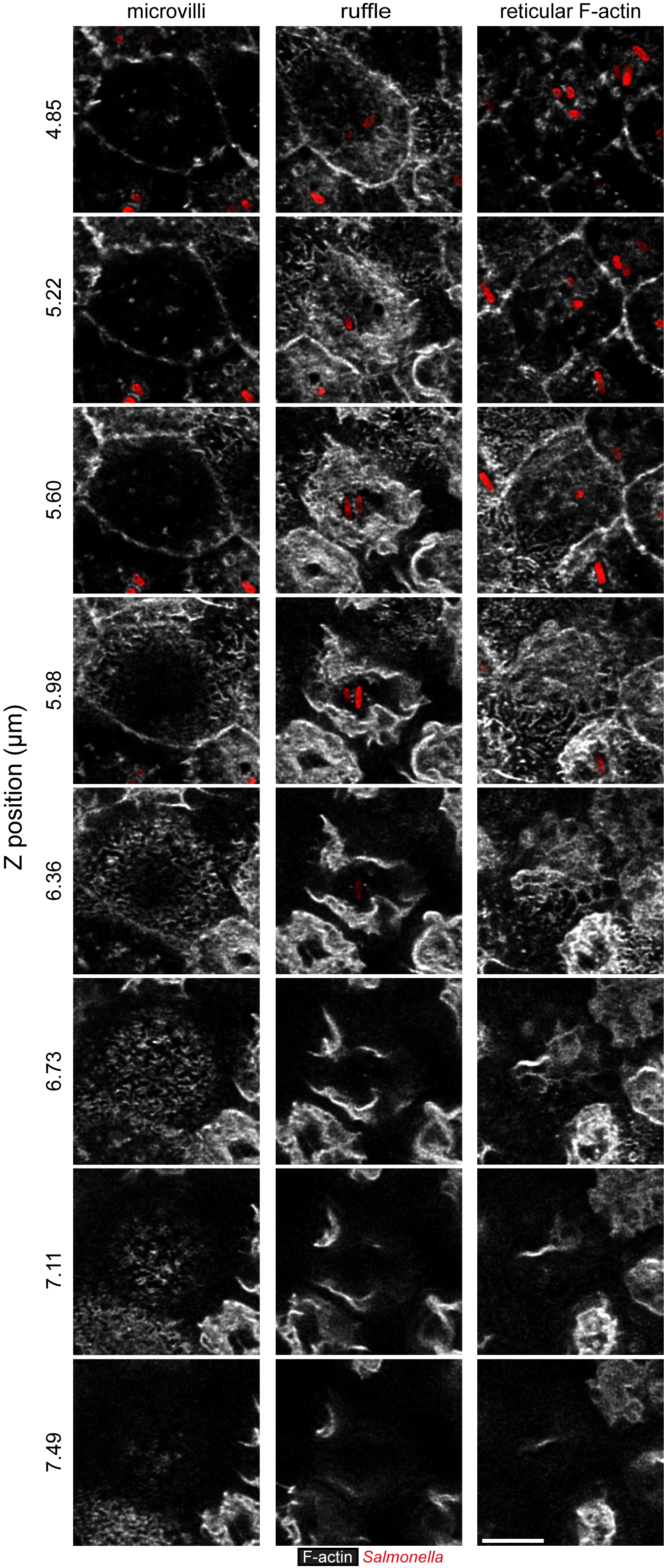

Supplement: Supplementary Figure 1 — Salmonella-induced F-actin reorganization of the apical side of polarized epithelial cells. Micrographs of Z-stacks for cells shown in Figure 1 are displayed. F-actin signals for reticular F-actin, microvilli and ruffles were only observed at the apical side of host cells. Scale bar, 15 µm. [file Image_1.tif]

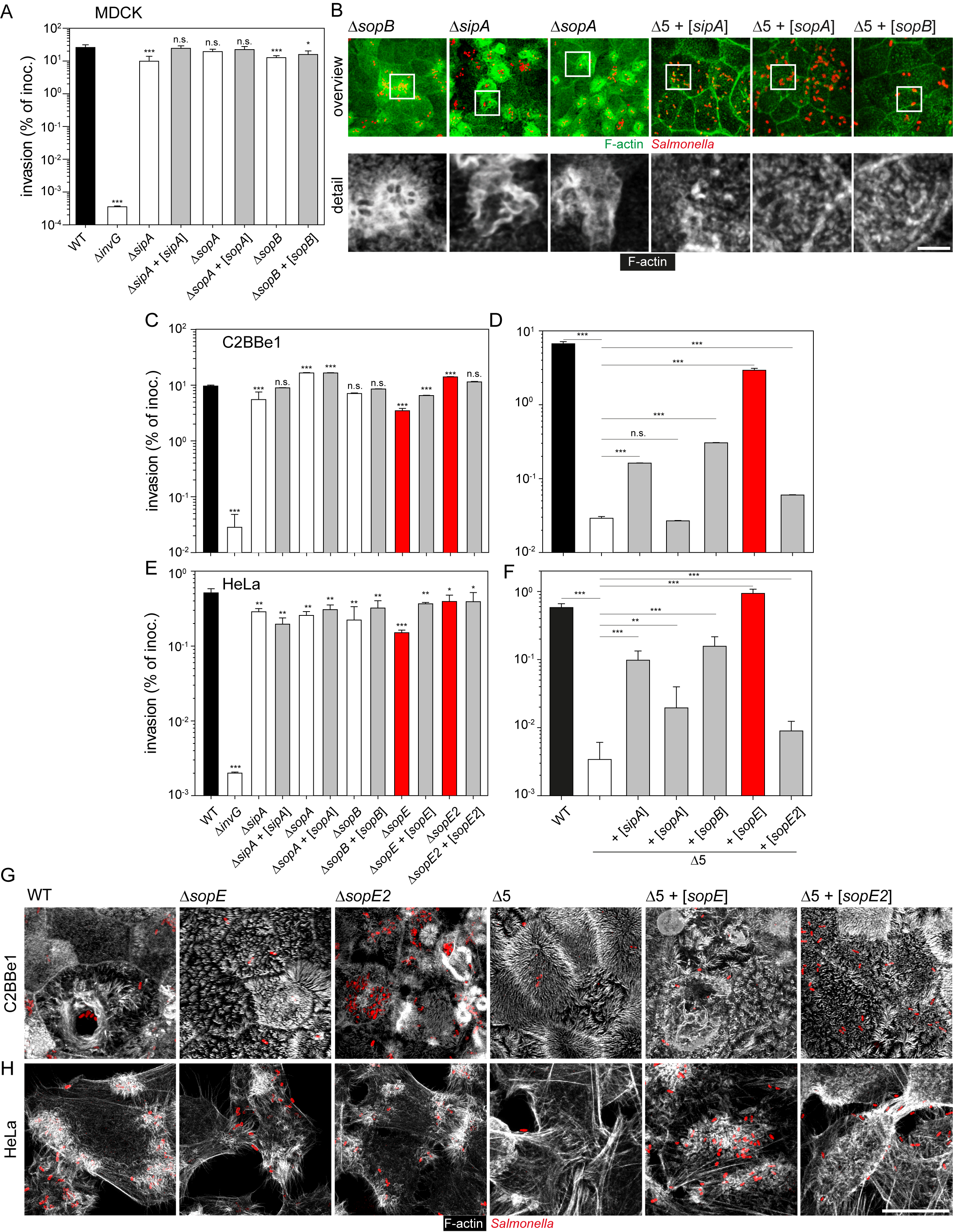

Supplement: Supplementary Figure 2 — SPI1-T3SS effector proteins have divergent effects on F-actin reorganization in polarized epithelial cells. Analyses of invasion of MDCK (A, B), C2BBe1 (C, D, G), or HeLa (E, F, H) cells were performed with various STM strains as described for MDCK. C2BBe1 were grown on transwell filter as indicated for TEER measurements. HeLa cells were seeded at 250,000 cells per well and infected the next day at MOI 50. Infection was performed as indicated with STM WT, invG, ΔsipA, ΔsopA, ΔsopB, ΔsopE, or ΔsopE2 strains, or strain Δ5, without or with complementation plasmids for sipA, sopA, sopB, sopE or sopE2. Levels of invasion were compared to WT (A, C, E) or strain Δ5 (D, F). Deletion of sipA and sopB reduced invasion (A) of MDCK cells, and affected morphology of membrane ruffles in MDCK cells (B). Complementation of sipA in strain Δ5 induces actin recruitment around the bacterial cell (B). Complementation of only sopB or sopA was not sufficient to induce changes in the F-actin cytoskeleton (B). Deletion of sopE decreases invasion of C2BBe1 (C) and HeLa cells (E). Complementation with sopE in strain Δ5 recovers invasion of C2BBe1 (D) and HeLa cells (F). B, G, H) Micrographs of infected cells were acquired by CLSM as described for Figure 1 . Scale bar, 20 µm. [file Image_2.tif]

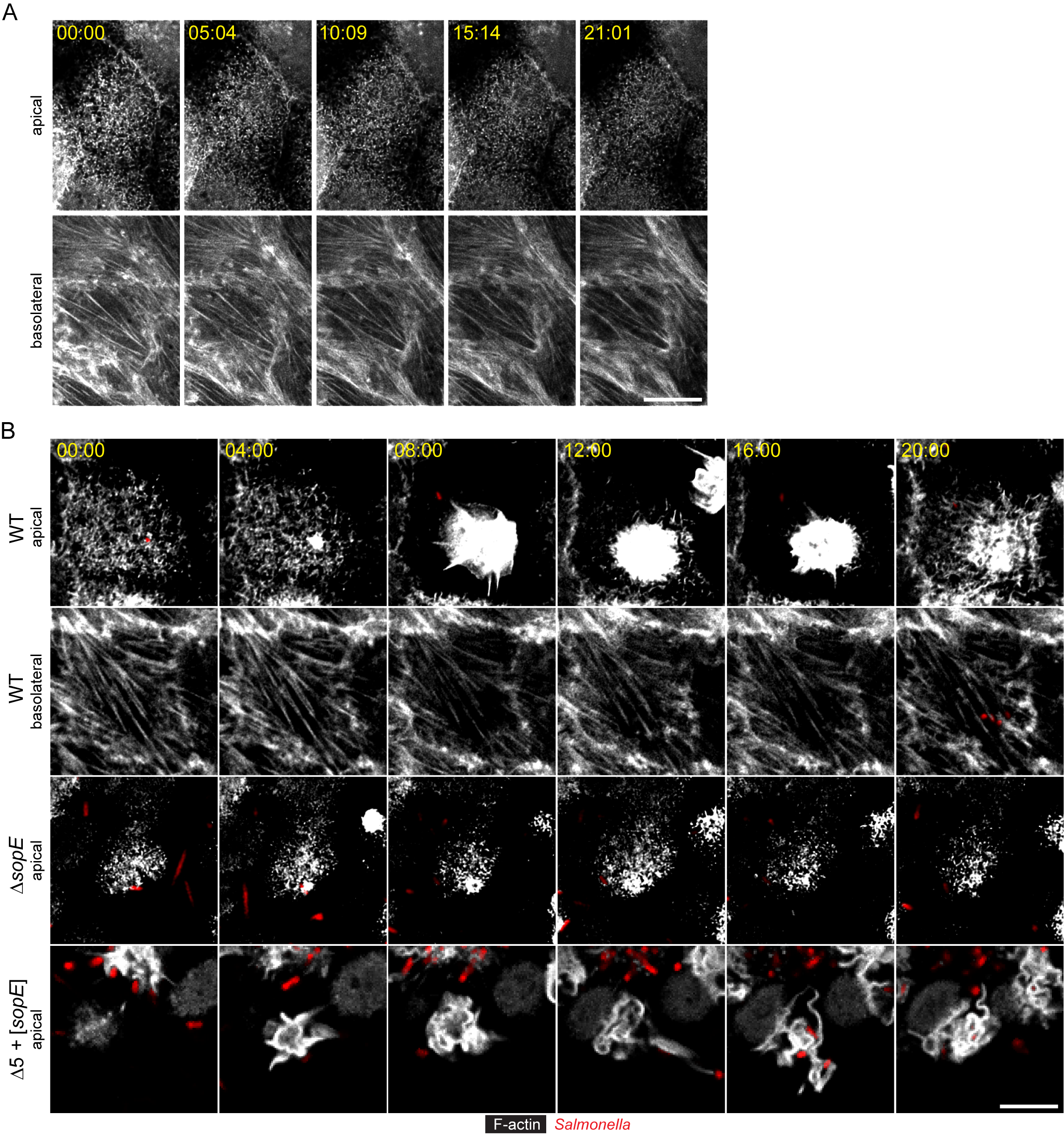

Supplement: Supplementary Figure 3 — MDCK cells transfected with Lifeact-eGFP allow observation of F-actin dynamics during Salmonella infection. (A) Microvilli dynamics at the apical side of MDCK cell. MDCK Lifeact-eGFP cells were seeded as described in Figure 2A and LCI was performed by SDM. Images from the apical and basolateral sides are shown and Movie 4 shows the time-lapse sequence for the apical side. (B) F-actin changes occur only at the apical, but not at the basolateral side of cells. The apical and basolateral sides of WT-infected cells from Figure 4 , as well as of cells infected by ΔsopE, Δ5 + [sopE] strains are shown. Scale bars, 10 µm (A), 15 µm (B). Time stamp, min:sec. [file Image_3.tif]

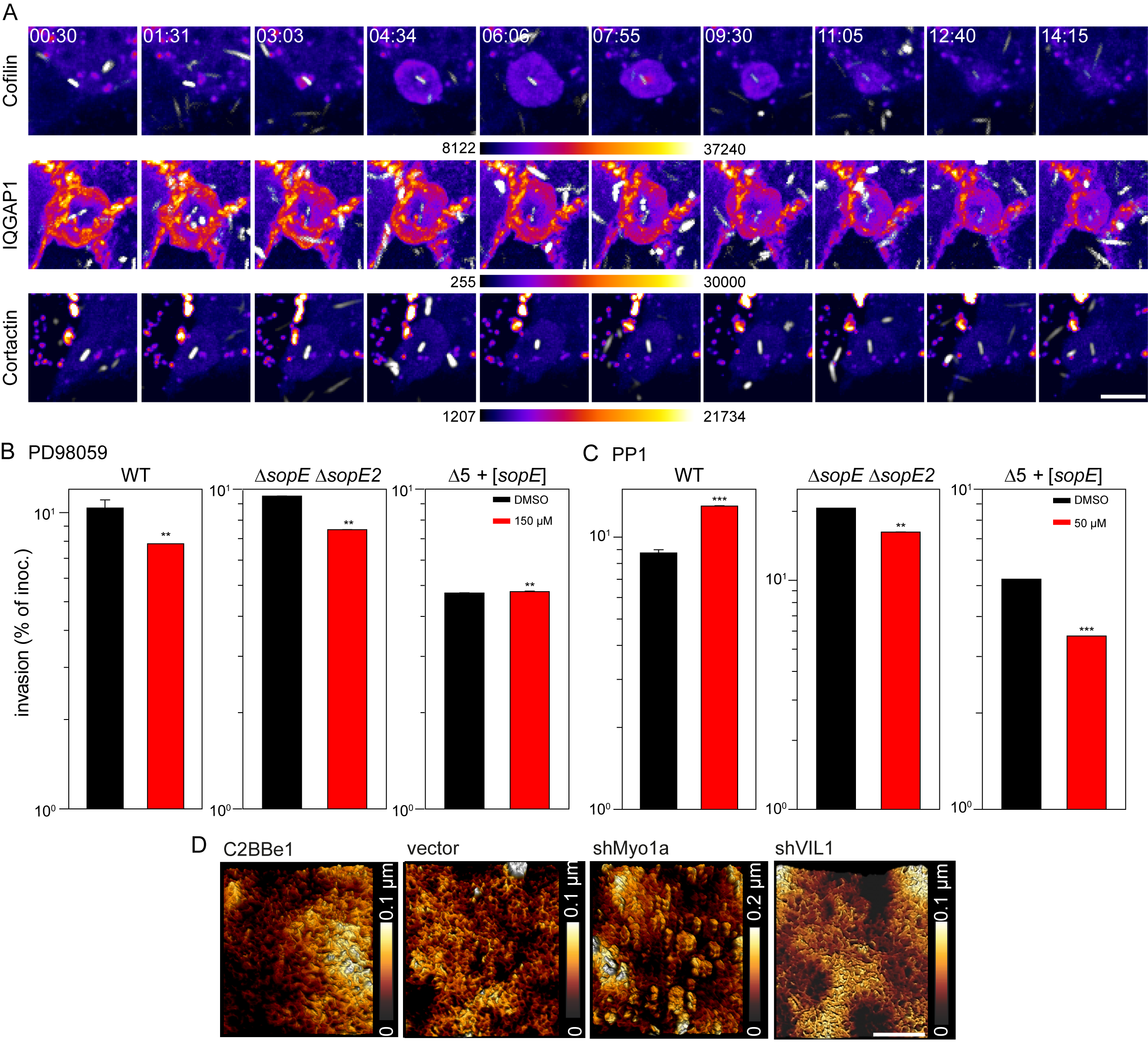

Supplement: Supplementary Figure 4 — IQGAP1, cortactin and cofilin are involved in the ruffle formation, but signaling pathways ERK1/2 and Src are not necessary. (A) The actin scaffold proteins IQGAP1 and cortactin, as well as the depolymerizing factor cofilin are recruited to the ruffle triggered by STM. MDCK cells were permanently transfected as stated in Material and Methods with plasmids carrying IQGAP1-eGFP, cortactin-RFP or cofilin-mCherry. Cells were infected as for Figure 4A . Images were continuously acquired by SDM for 30 min and MIP images are shown. Signal intensities are represented by pseudo-color scale (fire), the maximal and minimal intensities are given in the respective time series (time stamp, min:sec). See Movie 5 for time-lapse sequences. (B, C) ERK1/2 and Src kinases are not necessary for STM invasion of polarized cells. Inhibition experiments were carried out with the ERK1/2 kinase inhibitor PD98059 (B) and Src kinase inhibitor PP1 (C) or the solvent controls (DMSO). Inhibitors were added 30 min before infection and STM strains were used for infection as described for Figure 2A . Statistical analysis was performed comparing the levels of invasion of inhibited cells to DMSO-treated cells (negative control). (D) C2BBe1 cells were grown for 10 d and processed for AFM as described in Figure 1B . Scale bars, 4 µm (A), and 2 µm (D). [file Image_4.tif]
