# Supplementary material for: Manipulation of microvillar proteins during Salmonella enterica invasion results in brush border effacement and actin remodeling
Source: Front Cell Infect Microbiol. 2023 Mar 2;13:1137062. doi: 10.3389/fcimb.2023.1137062 (PMC10018140; doi:10.3389/fcimb.2023.1137062)
Supplement: Supplementary Table 1 — Oligonucleotides used in this study. [file Table_1.pdf]

**Table S 1. Oligonucleotides used in this study.**

| Designation | Sequence 5’-3’ |
|---|---|
| SopA-Red-Del13-Rev | caacgctgtgtcccttaattccatgcgggttgaggctggagtaggctggagctgcttcg |
| SopA-Red-Del13-For | ccagaccgtttttccataatgatgttgataaggaattctaattccggggatccgtcgac |
| SopE2-Red-Del-For | aaagtgtagctatgcatagttatctaaaaggagaactaccgtgtaggctggagctgcttc |
| SopE2-Red-Del-Rev | taattcatatggttaatagcactattgtatttactaccacatatgaatatcctccttag |
| SopA-Check | cctgccagataacatggtgaatt |
| SopE2-Red-Check-For | ctaaaaggagaactaccgtg |
| ProSicA-For-HIII | agcaagcttcaaaacccatcgccgttatg |
| ProSicA-Rev-EcoRI | agtgaattctgtcaccgactttgtagaac |
| SopA-For-XhoI | ttactcgagattttacatttcctgaacacgc |
| SopA-HA-Rev-XbaI | gcttctagattaagcgtagtctgggacgtcgtatgggtacgccccaggccagtggcagg |
| SopB-For-XhoI | ttactcgagtcacggtcttacttgtccggg |
| SopB-HA-Rev-XbaI | gcttctagattaagcgtagtctgggacgtcgtatgggtaagatgtgattaatgaagaaat |
| SopE-For-XhoI | taactcgagtacgctcaacgatcagctcac |
| SopE-HA-Rev-XbaI | gcttctagattaagcgtagtctgggacgtcgtatgggtagggagtgttttgtatatattt |
| SopE2-For-XhoI | taactcgagtacgctcaacgatcagctcac |
| SopE2-HA-Rev-XbaI | gcttctagattaagcgtagtctgggacgtcgTatgggtaggaggcattctgaagatactt |
| SipA-For-EcoRI | tgtgaattcctgcaaggataacagaagagg |
| SipA-HA-Rev-XbaI | gcttctagattaagcgtagtctgggacgtcgattgggtaacgctgcatgtgcaagccatc |
| MYO1A-1-a | cgcgtccccgctcaagaatcttcagcttcgttcaagagacgaagctgaagattcttgagctttttggaaat |
| MYO1A-1-b | cgatttccaaaaagctcaagaatcttcagcttcgtctcttgaacgaagctgaagattcttgagcgggga |
| VIL1-1-a | cgcgtccccgcacctttggaagcttcttcgttcaagagacgaagaagcttccaaaggtgctttttggaaat |
| VIL1-1-b | cgatttccaaaaagcacctttggaagcttcttcgtctcttgaacgaagaagcttccaaaggtgcgggga |
